# Topographic Modification of Semi-Crystalline Thermoplastic Polyetheretherketone (PEEK) using Argon Plasma and Femtosecond laser: A Lab-Based Evaluation

**DOI:** 10.12669/pjms.41.7.12185

**Published:** 2025-07

**Authors:** Abdullah Aljamhan, Fahad Alkhudhairy

**Affiliations:** 1 Abdullah Aljamhan Restorative Dental Sciences Department, College of Dentistry, King Saud University, Riyadh, Saudi Arabia; 2 Fahad Alkhudhairy Restorative Dental Sciences Department, College of Dentistry, King Saud University, Riyadh, Saudi Arabia

**Keywords:** Argon Plasma, Femtosecond laser, Polyetheretherketone, Surface roughness, Shear bond strength

## Abstract

**Objectives:**

To find out influence of advanced surface modification techniques (argon (Ar) Plasma, Femtosecond (FS) laser, and nano-hydroxyapatite (HA) coating) on the surface roughness (Ra) and shear bond strength (SBS) of resin luting cement to polyetheretherketone (PEEK).

**Methods:**

The lab-based comparative study followed a checklist for reporting in vitro study (CRIS) guidelines. The study was completed in two months (January 10, 2025, to March 10, 2025) at King Saud University. The study was conducted on sixty-four CAD CAM Vestakeep PEEK tooth-colored discs, divided into four groups (n=16) based on different conditioning methods SA (Group-1), Ar Plasma (Group-2), FS laser (Group-3), and nano-HA coating (Group-4). The Ra was evaluated using a profilometer, while topographical assessments were performed using scanning electron microscopy. Forty samples were subjected to resin cement luting, followed by artificial aging. A universal testing machine was utilized for SBS testing, and failure mode determination was performed using a stereomicroscope. Statistical analysis involved a one-way analysis of variance and Tukey’s Post Hoc test, with significance set at p=0.05

**Results:**

The highest Ra (1299.53 ± 0.015) and SBS (15.13 ± 0.11 MPa) values were observed in Group-3 (FS laser). Conversely, the lowest Ra (888.58±0.011) and bond strength (13.43±0.09 MPa) values were exhibited by Group-4 (Nano-HA coatings) specimens.

**Conclusion:**

The application of Argon Plasma and Femtosecond laser techniques has shown promise as viable alternatives for PEEK surface conditioning.

## INTRODUCTION

Since its introduction in 1978, polyetheretherketone (PEEK) has gained significant attention in prosthodontics, owing to its remarkable mechanical properties.^1^ However, the aesthetic limitations imposed by PEEK’s grayish-brown hue restrict its suitability for indirect restorations, especially in anterior regions where visual appeal is paramount.^2^ Additionally, prior investigations have revealed that the bonding of PEEK to dental structures using resin-luting cement yields inadequate shear bond strength (SBS), attributable to the material’s elevated surface tension, chemical inertness, and stable surface characteristics.^3^

Surface conditioning is a crucial step in mitigating this limitation, and offers improved mechanical outcomes.^4^ This process enhances the mechanical and biological properties of a material by altering its surface characteristics while preserving its inherent bulk properties.^5^ Conventionally sulfuric acid (SA) is used for the conditioning of PEEK. However, research indicates that 98% of SA are unsuitable for clinical applications owing to their highly corrosive properties.^6^ Additionally, substantial production of sulfur adversely affects human cells through DNA degradation.^7^

Plasma surface conditioning has emerged as an important technique in materials science.^8^ This method can be used to modify the material surface topography through processes such as ultra-purification, etching, and surface activation.^9^ Research has demonstrated that plasma employing argon (Ar) gas enhances the SBS of ceramic when used with resin-luting cement.^10^ In addition to plasma, various laser technologies have exhibited considerable potential in altering the surface properties of both dental tissues and materials, owing to their superior penetrative capabilities.^10^

Among the array of laser technologies, ultra-short-pulse lasers (UPL), particularly femtosecond (FS) lasers, have gained prominence as a leading surface modification technique owing to their ability to minimize thermal damage to treated surfaces.^11^ These lasers offer an advantage over conventional pulse lasers by delivering energy to the material in an extremely brief timeframe, thereby avoiding thermal diffusion.^12^ However, the efficacy of this technique as a conditioning protocol for PEEK, specifically regarding Ra and SBS with resin cement, remains unexplored and necessitates further investigation.

Recent progress in nanotechnology has enabled the practical application of nano-hydroxyapatite (nano-HA) in dental science, with particle dimensions ranging from 50 to 1000 nm. Previous research has demonstrated that these nanoparticle (NP) coatings create a uniform, slippery layer that exhibits sufficient durability and enhances the mechanical robustness of zirconia-based ceramics.^13^ Nevertheless, the effectiveness of nano-HA as a surface modification agent on the roughness average (Ra) and retentive bond strength of polyetheretherketone (PEEK) combined with composite materials remains unexplored in the current literature.

In this study, the effects of advanced surface conditioning techniques (FS laser, Ar Plasma, and Nano-HA coatings) on the Ra and SBS of PEEK in conjunction with resin cement were investigated. The investigators postulated that employing these state-of-the-art surface modifiers would lead to a substantial increase in PEEK’s Ra of PEEK compared to that of SA. Furthermore, it was anticipated that the application of the FS laser, Ar Plasma, and Nano-HA coatings would result in higher SBS values for PEEK with luting cement relative to the control.

## METHODS

The lab-based comparative study followed a checklist for reporting in vitro study (CRIS) guidelines. The study was completed in two months (10-Jan 2025 to 10^th^ March 2025) at King Saud University.

### Ethical Approval:

The research experiments conducted in this article were approved by the Ethical Committee of King Saud University under IRB number FC-99-911-25; dated January 1, 2025.

### PEEK discs preparation:

Sixty-four CAD CAM Vestakeep^®^ PEEK tooth-colored discs (Daical-Evonik, Tokyo, Japan) with a diameter of 5 mm and thickness of 3 mm were obtained by sectioning them from a PEEK cylinder using a slow-speed saw under constant water irrigation. These plates were then rooted in autopolymerizing acrylic resin (Acronstone, Cairo, Egypt), keeping the intaglio surface facing upwards. Polishing was performed using 120-, 400-, and 600-grit silica carbide papers under continuous water irrigation. Subsequently, an ultrasonic cleaner (Transsonic T700, Elma, Singen, Germany) filled with distilled water was used to immerse the discs for 10 min and then air dried.^14^

### Samples allocation and surface conditioning regime:

PEEK discs were allocated into four different groups based on the surface modification protocol used (n=16 each)

#### Group-1 (SA):

100 μL of 98% SA (RCI Labscan, Samutsakorn, Thailand) was smeared on the discs and left for 60 s. This was followed by rinsing with distilled water for 10 seconds.^15^

#### Group-2 (Ar-Plasma):

The PEEK discs were treated with Ar Plasma gas utilizing equipment (Surface-Engineering and Plasma Solution LTDA, Campinas, SP, Brazil) operating at a frequency of 13.56 MHz. Ar plasma gas was introduced into the chamber at a flow rate of 30 sccm. The pressure was maintained at 30 Pa and 500 V for 25 minutes.^16^

#### Group-3 (FS-laser):

FS laser (Mantis-Legend, Coherent) irradiation with a wavelength of 1026 nm was focused on the PEEK plates using a fused silica lens with a spot diameter of 20 μm. The laser parameters were set at a pulse duration of 220 fs, and a repetition rate of 100 kHz.

#### Group-4 (Nano-HA coating):

The nano-HA coating was prepared as a slurry consisting of 10 g of nano-HA powder (Merck, Germany) mixed with 50 mL distilled water. Polyvinyl alcohol (PVA; 1 g; Merck, Germany) was used to facilitate the binding of the solution. The suspension was heated on a magnetic stirrer at 100°C and a rotational speed of 1000 rpm for 60 s to achieve a homogeneous mixture. The discs were subsequently immersed in the slurry at a 45° angle for five seconds.

#### Ra analysis:

The Ra of the PEEK discs (n=5) was analyzed with a profilometer (Surftest-402, Mitutoyo, Kanagawa, Tokyo, Japan) equipped with a stylus gauge. The gauge was designed to move at a rate of 0.1 mm/s on a center of 2 mm designated for measurements. Three readings were obtained from three different points, and the mean was calculated^6^

### Topographical analysis:

The analysis was conducted using scanning electron microscopy (SEM) (JSM-5910LV, JEOL, Peabody, MA, USA). A disc from each conditioning Group-(n=1) was randomly selected and subjected to gold sputtering (MED 010, Balzers, Balzer, Liechtenstein) before examination under the SEM set at 15.0 kV at a working distance of 10 mm from the sample.

### Resin luting cement placement and Artificial aging:

Ten conditioned discs from each Group-were treated with Clearfil Ceramic Primer Plus (Kuraray, Tokyo, Japan) and allowed to air-dry. The dual-curing resin cement Panavia® V5 (Kuraray, Tokyo, Japan) was mixed and introduced into a silicone mold (3 mm and a thickness of 2 mm) positioned perpendicular to the discs. Following the meticulous elimination of surplus cement with a micro-brush, the cement was subjected to light curing from two sides for 20 s using an LED light-curing unit (Elipar S10, 3 M ESPE, St. Paul, MN, USA). All the specimens were subjected to artificial aging for 10,000 cycles in a thermocycler (Tae-won Tech, Incheon, Korea). The temperature was set in two distilled water baths at 5 and 55°C. The dwelling time in each bath was 30 s, with a transfer time of 5s.

### SBS testing and failure analysis:

PEEK and resin cement-bonded discs were firmly positioned on a universal testing machine (UTM) (EZ Test, Shimadzu Corp, Kyoto, Japan).The samples were exposed to a loading force using a metal fixture at a rate of 1.0 mm/min. The force required to debond each sample was recorded in megapascals (MPa). A stereomicroscope (MSV 330, Anyty 3R, Osaka, Japan) at 40x magnification was used to ascertain the type of failure. These modes were categorized into three types: cohesive, adhesive, and admixed.

### Statistical analysis:

To identify data normality, the Shapiro-Wilk test was used. InterGroup-comparisons between the study groups were performed using one-way analysis of variance (ANOVA) and Tukey’s post-hoc test.

## RESULTS

### Ra and SBS Assessment:

**Fig.1:**
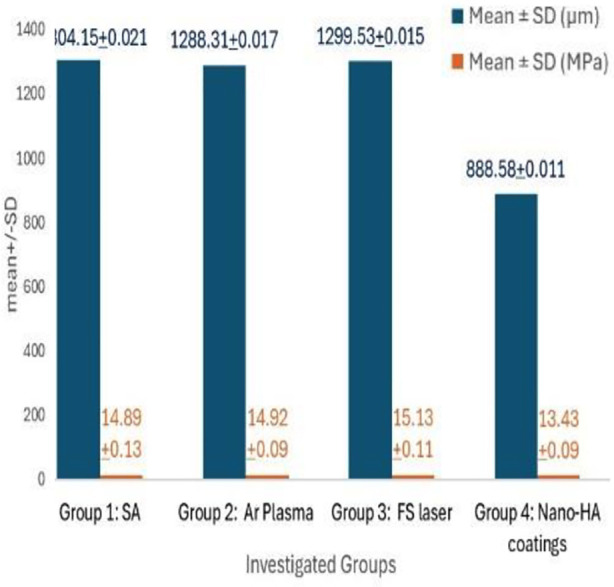
Ra and SBS of PEEK after applying various conditioners bonded to resin-luting cement.

Table-I displays the Ra values of PEEK after the application of the various conditioners. The highest Ra scores were observed in Group-3 (FS laser) (1299.53±0.015) pretreated discs. The lowest outcomes were exhibited by Group-4 (nano-HA coatings) (888.58±0.011). InterGroup-comparison analysis revealed that Group-1 (SA) (1304.15±0.021), Group-2 (Ar Plasma) (1288.31±0.017), and Group-3 samples displayed no significant difference in their Ra scores. (p>0.05).Table-II displays the SBS of PEEK for resin-luting cement after applying various conditioners. The strongest bond was observed in Group-3 (FS laser) (15.13±0.11 MPa). The weakest bond was exhibited by Group-4 (nano-HA coatings) (13.43±0.09 MPa). InterGroup-comparison analysis unveiled that Group-1 (SA) (14.89±0.13 MPa), Group-2 (Ar Plasma) (14.92±0.09 MPa), and Group-3 treated samples displayed no significant difference in their bond strength scores. *(p>0.05)* (Fig.1).

**Table-I:** Ra of PEEK after applying various conditioners.

Investigated groups	Mean ± SD (µm)	p- value!
Group-1: SA	1304.15±0.021^*^	*<0.05*
Group-2: Ar Plasma	1288.31±0.017^*^	
Group-3: FS laser	1299.53±0.015^*^	
Group-4: Nano-HA coatings	888.58±0.011^π^	

! ANOVA Sulfuric acid (SA), Femtosecond lasers (FS), Argon (Ar), Hydroxyapatite, Different superscript characters denote statistically significant differences (Post Hoc Tukey Multiple Comparison Test).

**Table-II:** SBS of PEEK bonded to resin luting cement after applying various conditioners.

Investigated groups	Mean ± SD (MPa)	p-value!
Group-1: SA	14.89±0.13^*^	*<0.05*
Group-2: Ar Plasma	14.92±0.09^*^	
Group-3: FS laser	15.13±0.11^*^	
Group-4: Nano-HA coatings	13.43±0.09^π^	

! ANOVA Sulfuric acid (SA), Femtosecond lasers (FS), Argon (Ar), Hydroxyapatite, Different superscript characters denote statistically significant differences (Post Hoc Tukey Multiple Comparison Test).

### Fracture pattern analysis:

**Fig.2:**
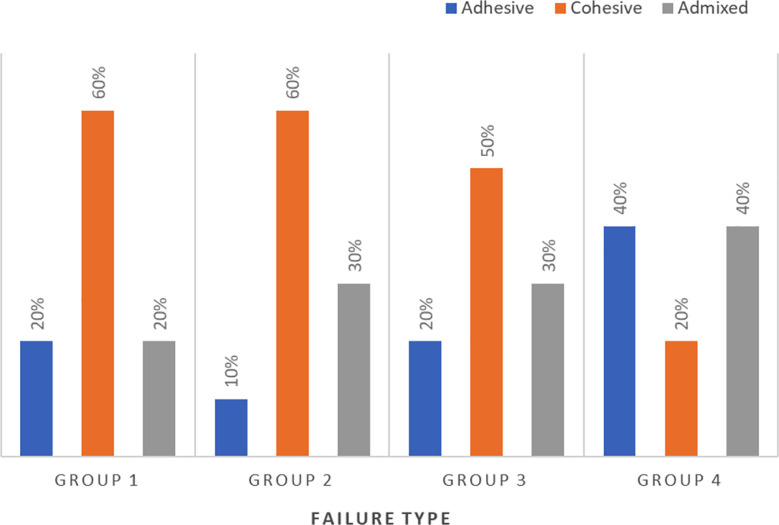
Modes of failure distribution in different experimental groups.

Debonded surfaces indicated an adhesive failure mode predominantly in the nano-HA coating group. However, the FS laser-, Ar Plasma, and SA-conditioned surfaces presented mostly admixed and cohesive failures (Fig.2).

## DISCUSSION

This investigation was based on the premise that the Ra of PEEK following treatment with modern surface modifiers substantially exceeded that of SA. Additionally, it was postulated that the SBS between PEEK and luting cement would be enhanced when subjected to conditioning with FS laser, Ar Plasma, and Nano-HA coatings relative to the control group. The experimental findings revealed that specimens treated with FS and Ar plasma demonstrated no statistically significant variance from SA-pretreated PEEK discs in terms of Ra and SBS, thereby lending partial acceptance to both the proposed hypotheses.

Achieving sufficient Ra is crucial for ensuring adequate mechanical retention during bonding.^17^ However, PEEK’s inherent high strength of PEEK poses challenges to surface roughening techniques owing to its elevated hardness and strength.^14^ The results indicated that Ar plasma and FS laser treatments yielded outcomes comparable to those of the control. The literature suggests that plasma surface treatment can effectively modify polymer surfaces, inducing various physical and chemical alterations to the surface characteristics of the material.^18^ This phenomenon can be attributed to the transformation of the non-polar characteristics of the PEEK substrate into a polar state when exposed to low-temperature plasma, resulting in surface roughening.^19^ Additionally, this process forms a dense cross-linked layer on the surface, enhancing the bonding interface through the improved mechanical interlocking of the resin, thereby augmenting bonding performance.^19^ SEM imaging of the Ar-plasma-treated sample corroborated these findings, revealing numerous grooves and cracks on the surface (Fig.3A). The enhanced Ra and SBS observed after FS laser application on PEEK discs aligns with Kara and the coauthor’s laboratory analysis of zirconia ceramics, which identified FS laser irradiation as an efficient surface conditioning method for Ra enhancement.^20^ SEM images of FS laser-conditioned PEEK discs further supported this conclusion, displaying a series of pronounced horizontal grooves that improve resin cement retention and significantly enhance bond strength (Fig.3B). These findings were consistent with those of previous in vitro studies.^20^,^21^

**Fig.3A:**
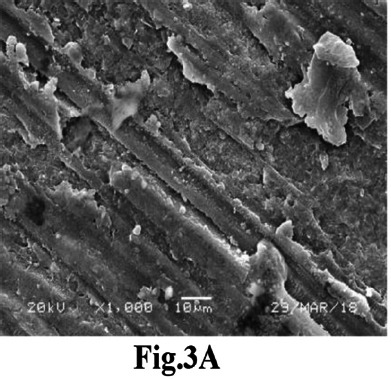
SEM image of the Ar-plasma-treated samples revealing numerous grooves and cracks on the surface

**Fig.3B:**
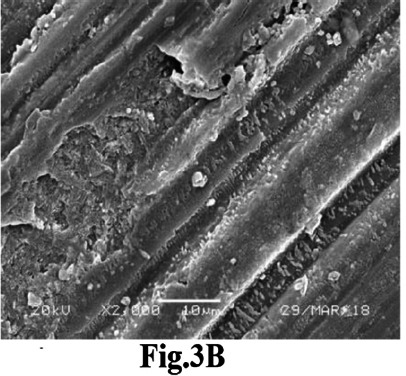
SEM image of PEEK disc treated with FSL displaying pronounced horizontal grooves that improve resin cement retention and enhance bond strength

For an extended period, nano-HA coatings have been employed in implant dentistry to improve the adhesion of surrounding cells to implant surfaces.^22^ PEEK discs pretreated with the nano-HA coating showed decreased Ra scores and diminished bond strength with resin cement. This finding contradicts the results of the recent study by Atri et al.^23^ Based on the SEM images, the authors suggest that the nano-HA coating did not enhance Ra when applied to PEEK, potentially explaining the lower SBS observed (Fig.3C). However, available data is limited, necessitating further research in this area. The analysis of the failure patterns predominantly indicated an adhesive failure mode in the nano-HA coating group. However, the FS laser-, Ar Plasma, and SA-conditioned surfaces presented mostly admixed and cohesive failures. This study aligns closely with the results reported by Atsu et al.^24^ which identified adhesive failures occurring at lower bond strength values, while higher bond strengths were associated with mixed and cohesive failures.^24^,^25^ The present study highlights the alternate methods (Argon Plasma, laser, and Nano-HA coatings) to improve surface topography and adhesive strength of PEEK to improve clinical outcomes and treatment prognosis in patients.

**Fig.3C:**
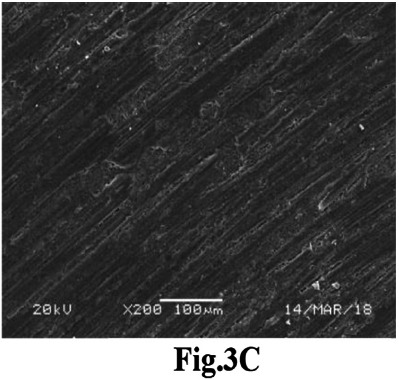
SEM image of PEEK surface coated with nano-HA displaying no surface roughness grooves or projections.

### Limitations

The current study has several limitations, including its in vitro design, which necessitates cautious extrapolation of the results. The study employed only one set of FS laser parameters and a single HA concentration, potentially limiting the scope of the outcomes. Future studies should explore various parameters and concentrations. Also, other mechanical testing of flexural, compressive, and tensile strength needs to be assessed. Further laboratory experiments and clinical studies are necessary to validate the conclusions drawn from this analysis.

## CONCLUSION

FS lasers and Ar plasma have shown promise as alternatives to sulfuric acid for PEEK surface conditioning. These methods yield similar Ra and bond strength results while avoiding tissue-damaging properties associated with caustic substances.
